# Texas State Medical Association

**Published:** 1898-04

**Authors:** 


### Society Notes.

### TEXAS STATE MEDICAL ASSOCIATION.

### Preliminary Announcement and Program of the Thirtieth Annual Meeting, Tuesday, Wednesday, Thursday and Friday, April 26th, 27th, 28th and 29th, 1898.

TURNER HALL, HOUSTON.

Committee of Arrangements.—Joseph A. Mullen, chairman; Donald McKay, secretary; J. W. Scott, W. R. Eckhardt, R. W. Knox, E. P. Daviss, S. C. Red, George Larendon, R. T. Morris, E. B. Jackson, J. B. Massie, W. N. Shaw, J. R. Stuart, F. B. King, Max Urwitz.

TRANSPORTATION.

Owing to the energy and zeal of the above committee, the railroads offer a rate which is unprecedented so far as the Medical Association is concerned. The following is an explanatory circular of the Gulf, Colorado & Santa Fe Railway. The same rates have been allowed by the other roads:

GALVESTON, April 2, 1898.

*Supplement No. 1 to Special Order No. 2931,—State Medical Association; Houston, Texas, April 26 to 29, 1898.*

All Agents.—Referring to the above special order, quoting rate of one and one-third fares:

This is to advise you that rates, instead of being made on this basis, will be made on the distance plan, as follows:

From stations within 75 miles of Houston, rate of one and one-third fares; from stations between 75 and 100 miles distant, rate of $3; from stations over 100 miles distant, rate of one fare for the round trip.

Dates of Sale.—Sell April 25 and 26, 1898.

Limit.—Limit all tickets to leave Houston not later than train 15, leaving Central depot at 6:55 p. m., April 30, 1898.

Agents will note that this cancels limit of May 5, as quoted in the original order.

W. S. KEENAN,
General Passenger Agent.

### HOTEL RATES.

Ample hotel accommodations are available. Reduced rates as follows: Capitol Hotel, $2 per day; Lawlor House, 2; Hutchings House, $1.50; Bristol House, $1.50, and private boarding houses, $1.

### PRELIMINARY PROGRAM.

The session will begin Tuesday morning, April 26, at 11:30 a. m., at Turner Hall, corner Prairie and Caroline streets. Members of the Arrangement Committee will be at the hall by 9 a. m., at which time the books will be open for registration. Members of the local societies can join the State Association by presenting certificates signed by the President and Secretary. Other applicants will be required to exhibit diploma or satisfactory evidence of graduation to the Judicial Council. Annual dues, $5, and initiation fee, $2.50, must accompany all applications. Badges and programs will be distributed after registration. Titles of papers appearing herein will take precedence of others subsequently offered.

ORDER OF EXERCISES.

Call to order by Chairman Committee of Arrangements, Joseph A. Mullen, Houston.

Invocation, Rev. Henry D. Aves.

Welcome, Hon. Samuel A. Brashear, Mayor.

Welcome, representing the Citizens of Houston, Hon. J. W. Jones.

Welcome, representing the Medical Profession of Houston, Joseph A. Mullen.

Response by the President, Bacon Saunders, Fort Worth.

Roll Call.

Reading Minutes of Previous Meeting.

Secretary's Annual Report, H. A. West.

Treasurer's Annual Report, J. Larendon.

President's Annual Message and Recommendations, Bacon Saunders.

The President's Address and Annual Oration will be delivered on Thursday evening at 8 o'clock, at Turner Hall, and will be followed by an informal reception in the garden adjoining. Music and dancing.

Boat excursion down Buffalo Bayou from Clinton.

Reception Wednesday afternoon from 5 to 7 o'clock at residence of Dr. D. F. Stuart, Texas avenue and San Jacinto street.

SECTIONS.

Morning Session, 9 o'clock. Afternoon, 2:30 o'clock. Night Session, if necessary, beginning at 7:30.

GENERAL MEDICINE.

1. Report of Chairman: A. B. Gardner, Bellville.

2. Differential Diagnosis Between Yellow Fever and Dengue, with Some Account of the Epidemic in Texas of 1897: H. A. West, Galveston. Discussion opened by R. T. Morris, Houston.

3. Yellow Fever: J. D. Burch, Aurora.

4. The Decadence of the Drug Treatment of Disease: Frank B. King, Houston. Discussion opened by B. F. Calhoun, Beaumont.

5. Hydrotherapy: J. H. McCracken, Mineral Wells.

6. A Pathological Problem—"Fever; What is It?" J. M. Fort, Paris.

7. Some Reports Concerning Hypnotism: Matthew M. Smith, Austin. Discussion opened by Edgar D. Capps, Fort Worth.

J. S. WOOTEN, Secretary, Austin.

### SECTION ON OBSTETRICS AND DISEASES OF CHILDREN.

1. Report of Secretary: T. L. Kennedy, Galveston.
2. Eclampsia—Etiology; W. F. Starley, Jr., Galveston.
3. Symptoms: T. W. Shearer, Wallisville.
4. The Eye Symptoms: Vard H. Hulen, Galveston.
5. Treatment: W. R. Blailock, McGregor.
6. Congenital Deformity—Report of Cases: E. J. Neathery, Van Alstyne.

### SURGERY—PART I.

1. Report of Chairman: B. E. Hadra, San Antonio.
2. Surgical Treatment of Empyema, etc.: T. W. Shearer, Wallisville.
3. Some Observations on Fractures of the Skull: J. R. Stuart, Houston. Discussion opened by P. M. Raysor, Bosque.
4. Subcutaneous Arterial Ligation: T. J. Pugh, Hearne. Discussion opened by Matt M. Smith, Austin.
5. Report of Two Cases of Appendicitis: W. R. Blailock, McGregor. Discussion opened by W. G. Jameson, Palestine.

### PART II.

The principal topic of this Section will be a discussion of "Fractures and Dislocations of the Foot, including the Ankle Joint; with Surgical and Orthopedic Treatment of Same." In order to have a full representation of the matter, the subject has been divided into five parts; each division to be introduced by different gentlemen. It is hoped that an ample discussion of this practical and important subject will be evoked, and the members of the Society will fully enter into the debate by giving their experiences and report of cases.

6. Fractures Above and Through the Malleoli: R. T. Morris, Houston.
7. Dislocations in the Ankle and Tarsal Joints: J. E. Thompson, Galveston.
8. Fracture of Tarsal Bones: J. E. Gilcreest, Gainesville.
9. Orthopedic Treatment of Badly Healed Fractures and Dislocations: M. M. Edmondson, San Antonio.

10. Surgical Treatment of Badly Healed Fractures of the Ankle and Tarsal Joints: C. A. Smith, Tyler.

DISCUSSION.

J. R. STUART, Secretary, Houston.

STATE MEDICINE AND PUBLIC HYGIENE.

1. Report of Chairman: S. F. White, Terrell.
2. How to Reduce the Death Rate and Number of Defective Population: A. N. Denton, Austin.
3. The Practice of Medicine Losing Caste in the Eyes of the Public: F. M. Davis, Houston.
4. The Prevention of Tuberculosis: Matthew M. Smith, Austin. Discussion opened by S. C. Red, Houston.
4. The Prevention of Tuberculosis: Matthew M. Smith, Austin. Discussion opened by S. C. Red, Houston.

S. E. Hudson, Secretary, Austin.

GYNECOLOGY.

1. Report of Chairman: H. K. Leake, Dallas.
2. Some Thoughts on Cancer of the Uterus: Thos. J. Crofford, Memphis, Tenn.
3. Surgical Treatment of Retro-Displacements of the Uterus—Report of Cases: S. M. Jenkins, Summer's Mills.
4. Operation far Removal of Right Tube, Ovary and Appendix: B. F. Kingsley, San Antonio.
5. Restitution of the Perineal Body: A. C. Scott, Temple.
6. Report of a Case: Thos. B. Dorris, Grapevine.
7. Conservative Treatment of Appendages: S. F. King, Sherman.
7. Report of a Case of Vicarious Menstruation: Joe D. Becton, Greenville.
9. A Study of Lacerations of Parturition, with Especial Reference to Recognition and Time of Repair: J. M. Coble, Dallas.
10. Report of Case and Presentation of Specimen: B. F. Kingsley, San Antonio.

S. F. King, Secretary, Sherman.

OPHTHALMOLOGY AND OTOLOGY.

1. Report of Chairman: R. F. Miller, Sherman.
2. The Relation of Our section to the State Medical Association: Vard H. Hulen, Galveston.

3. Some Ophthalmological Don'ts: S. L. Terrell, Dallas.

4. Early Operation in Pterygium: W. R. Thompson, Fort Worth.

5. Extract of Supra-Renal Capsule as an Adjuvant to Cocaine in Minor Operations on the Eye and its Appendages: J. A. Mullen, Houston.

6. Glaucoma—Report of a Case: R. H. Chilton, Dallas.

7. Superficial Keratitis—Malaria as an Ætiological Factor: F. D. Boyd, Fort Worth.

8. The Evolution of the Artificial Pupil: E. P. Daviss, Houston.

9. Notes from the Record Books of the State Institution for the Blind and the Texas Eye, Ear and Throat Hospital: H. L. Hilgartner, Austin.

10. Plastic Operations About the Eye: J. O. McReynolds, Dallas.

11. Chronic Suppurative Otitis Media: R. E. Moss, San Antonio.

12. Indications for Enucleation of the Eye-Ball: J. O. McReynolds, Dallas.

13. Transplantation of the Rabbit's Cornea to the Eye of Man: R. F. Le Mond, Denver, Col.

The extraordinary railroad rates obtained by the Committee of Arrangements, the above interesting list of papers, together with the local attractions, will doubtless insure a large attendance at Houston. The presence and co-operation of every regular and reputable practitioner in the State is urgently solicited. There are many matters of importance to come up which will require the united action of the profession to accomplish. County and district societies wishing to affiliate with the State Association are requested to send representatives with list of officers and members.

The Committee of Registration of the American Medical Association will be governed in accepting certificates of delegates by the lists of secretaries of each State society, i. e.: no county society not in affiliation with the State organization is entitled to representation in the American Medical Association. Officers of county societies please take notice. Each county society in affiliation with the State Association is required to pay $5 annually into the treasury of the latter.

H. A. WEST, Secretary.

HOUSTON, TEXAS, March 30th, 1898.

*My Dear Doctor:* The Committee of Arrangements desire to call your attention to the meeting of the Texas State Medical Association, Tuesday, April 26, 27, 28, 29, 1898. The program has been arranged with two objects in view: your comfort and pleasure.

Special rates have been made with the following hotels: Hutchins House, $1.50; Bristol Hotel, $1.50; Capitol Hotel, $2, and Lawlor House, $2. These rates will be given to doctors and their families. Private boarding houses, $1 per day.

Wednesday, April 27th, 2 p. m., a special train will carry you from Houston to Clinton, and thence down Buffalo Bayou on boats. Refreshments, music, etc.

Thursday, 28th, 5 p. m., immediately after adjournment of the convention, illuminated electric street cars will be in waiting in front of the hall to take you over thirty-six miles of street railway to all parts of our city.

Thursday, 28th, p. m., after the President's address, refreshments will be served in garden adjoining Turner Hall. Music and dancing will follow in the hall later. The committee will also be pleased to give you cards of invitation to the Houston Club, Elk Club, and Houston Light Guard Club.

The railroads coming into Houston will give you a one-fare rate for round trip, excursion tickets, no certificate—this is the cheapest transportation our Association has ever had.

Now, doctor, your committee have fully arranged for your pleasure and comfort, so attend; bring your wife and friends is the desire of

Yours truly,

JOSEPH A. MULLEN, Chairman.

| | |
|---|---|
| J. W. SCOTT, | W. R. ECKHARDT, |
| R. W. KNOX, | E. P. DAVISS, |
| S. C. RED, | GEORGE LARENDON, |
| R. T. MORRIS, | E. B. JACKSON, |
| J. B. MASSIE, | W. N. SHAW, |
| J. R. STUART, | F. B. KING, |
| MAX URWITZ | DONALD MCKAY, Sec. |

Committee.

N. B. The one fare round trip tickets will be on sale one day only, the 25th day of April. Delegates should secure their tickets on that date to get the benefit of the one fare rate.

